# The Dissector’s Manual of Practical and Surgical Anatomy

**Published:** 1856-11

**Authors:** 


*The Dissector's Manual of Practical and Surgical Anatomy.* By ERASMUS WILSON, F.R.S. &c. The Third American, from the Last Revised London Edition, Illustrated with One Hundred and Fifty-Four Wood Engravings; Edited by WM. HUNT, M.D. Demonstrator of Anatomy in the University of Pennsylvania. Philadelphia, Blanchard & Lea.

This is an excellent work of the kind; one of the best that can be placed in the hands of students. Its numerous and accurate engravings add much to its value. It is already well known to the profession.

For sale by Keen & Lee, Chicago. J.

